# Beyond Referral: Symptoms Still Matter in Severe Aortic Stenosis

**DOI:** 10.1016/j.shj.2026.100823

**Published:** 2026-02-17

**Authors:** Nicholas Spetko, Jordan B. Strom

**Affiliations:** a Division of Cardiology, Richard A. and Susan F. Smith Center for Outcomes Research in Cardiology, Beth Israel Deaconess Medical Center, Boston, Massachusetts; b Division of Cardiology, Beth Israel Deaconess Medical Center, Boston, Massachusetts

Aortic stenosis (AS) is the most common valvular disease in developed countries with moderate to severe AS identified in roughly 3% of the general population^1^^,^^2^ and it is increasing in prevalence with the aging of the population.^3^ It has been more than 20 years since Alain Cribier performed the first-in-human transcatheter aortic valve replacement (TAVR) in a 57-year-old with cardiogenic shock in the setting of severe AS and left ventricular dysfunction.^4^ In the last 2 decades, TAVR has revolutionized AS care, overtaking surgical aortic valve replacement (AVR) worldwide in 2019 as the dominant form of valve intervention, with technological advancements making TAVR safer^5^ and increasingly available.^6^

Despite increasing availability of TAVR,^6^ severe symptomatic AS remains undertreated, even at top medical centers, with nearly 1/3 of high-gradient symptomatic severe AS patients not receiving treatment^7^^,^^8^ despite having a 2% increased relative risk of mortality per week on the waitlist for a procedure.^9^^,^^10^ While reasons for this undertreatment burden are multifactorial, prior work has demonstrated that physicians underrecognize symptoms of AS, often failing to attribute these to a patient’s AS, and overestimate contemporary risks of intervention.^11^^,^^12^ As the diagnosis of AS becomes more complex with disease definitions stratified by left ventricular function, flow state, and valvular gradients,^13^ referring physicians may struggle to interpret the urgency of the clinical scenario, with evidence that those treated by noncardiologists or with low-flow, low-gradient AS may receive AVR at lower rates.^7^^,^^8^^,^^14^

In this setting, several randomized trials^15^, ^16^, ^17^ have suggested that patients with severe asymptomatic AS may benefit from AVR, calling into question the role of symptoms in the modern era. Moreover, increasing attention has been paid to the role of the echocardiographer in tying a diagnosis of severe AS to clinical recommendations for referral to the heart valve team (HVT).^18^ In the recently published DETECT AS (Electronic Provider Notification to Facilitate the Recognition and Management of Severe Aortic Stenosis) cluster randomized clinical trial,^19^ clinicians were randomized to receipt of an electronic notification of the diagnosis of severe AS, and this notification resulted in increased rates of AVR, lessened sex and age disparities in AVR use, and improved survival. As a result of such evidence, American Society of Echocardiography guidelines for standardized reporting of transthoracic echocardiography (TTE) now permit text embedded in the TTE report suggesting HVT referral in appropriate patients.^18^ In this setting, where patients may find themselves routed to the HVT solely based on their TTE measurements, one may ask the question, do symptoms still matter?^20^

In this issue of *Structural Heart,* Offen et al.^21^ offer some answers. The authors examine referral patterns to the HVT at a single center in Canada after implementation of an automatic TTE report prompt, encouraging HVT referral in those with moderate to severe AS. Of 142 patients with severe AS and 201 with moderate AS followed for a median of 226 days, only 61% and 22% of patients, respectively, were referred to HVT. During the same follow-up period, nonreferred patients experienced a higher all-cause mortality (19.6% vs. 2.3%), mirroring results from other settings.^7^^,^^11^ Despite all providers for these patients receiving an automated text prompt, which also populated the electronic medical record, referral rates remained low and similar to other centers.^7^^,^^8^ Of nonreferred patients who died, nearly half had no compelling reason for lack of referral, and 75% of nonreferred patients had New York Heart Association class II or III symptoms. Over half reported being managed under a “watchful waiting” strategy despite meeting Class I indications for AVR. Perhaps most concerning, nearly a third of nonreferred patients were not aware of their AS diagnosis.

While not entirely translatable to non-Canadian health systems where practice may differ, the article offers a stark but unmistakable point: the under-recognition of AS symptoms in clinical practice by both providers and patients themselves remains a critical issue. In the EARLY-TAVR (Transcatheter Aortic-Valve Replacement for Asymptomatic Severe Aortic Stenosis) trial (NCT03042104), exercise tolerance testing identified occult symptoms in nearly 15% of patients deemed by their screening physicians to be asymptomatic.^17^ Even despite this screen, nearly half (45.3%) of those that were randomized to the clinical surveillance arm of the trial ultimately developed symptoms within 6 months, often in a sudden and unpredictable fashion, increasingly being termed “acute heart valve syndrome” to draw attention to its acuity and similarity to other diseases (e.g., heart failure) where decompensation is part of the disease course. What’s more, patients who developed symptoms within this 6-month period were at higher risk for adverse outcomes after TAVR,^22^ similar to findings from other studies where symptom burden was associated with worse outcomes,^23^ suggesting urgency in identifying and treating these patients early.

There are several possible reasons for symptom under-recognition in this setting that deserve attention. First, the slowly progressive nature of AS may cause patients to unknowingly reduce their physical effort, and thus any symptom assessment is incomplete without also asking patients about their physical activity.^24^ Second, the primary tool for symptom assessment, that is exercise tolerance testing,^13^^,^^25^ may still be considered too high risk by some physicians due to prior contraindications in those with severe AS or may not be feasible for patients with orthopedic issues or peripheral arterial disease. Third, symptom assessment, despite still being important for risk stratification and early triage of patients for AVR, is not standardized across providers, contributing to the variable practice observed in multiple clinical settings. If providers are not aware of their own patient’s symptoms, how can we expect patients to understand the urgency of their disease?

Beyond referral to the HVT, the path forward requires reconsidering how we identify AS-related symptoms in practice through a structured, multimodal approach. First, exercise tolerance testing remains an underutilized tool in qualifying patients, currently used in <5% of those with severe AS.^13^^,^^25^ Increasing efforts should be focused on understanding reasons for this gap in utilization and the ability of other measures (e.g., wearable health data) to provide such functional status information. Second, existing validated symptom questionnaires (e.g., the Kansas City Living with Cardiomyopathy Questionnaire^26^) paired with wearable health data could be used to identify patients with subtle symptoms or those whose daily physical activity is insufficient to provoke symptoms. Third, novel artificial intelligence–aided tools intended to be used to identify those with heart failure,^27^ could be repurposed to identify patients with subtle symptoms. Overall, using such tools in a combined hierarchical fashion to identify symptoms requires validation and further prospective testing but holds promise for a structured and standardized way of making sure AS symptoms are not missed by patients or their providers.

While expanded indications for AVR in asymptomatic patients with severe AS offer an offramp for those in whom symptom assessment is equivocal or challenging, we should not be too quick to dismiss the continued importance of symptoms to the patients who have them. As Offen et al. demonstrate,^21^ improving symptom recognition will remain paramount in the foreseeable future to inform triage and align patient and clinician priorities, putting patients where they deserve to be, front and center as stewards of their own care.

## Funding

J.B. Strom reports research grants from the 10.13039/100000050National Heart, Lung, and Blood Institute (1R01HL169517, 1R01HL173998). N. Spetko is supported by the 10.13039/100000050National Heart, Lung, and Blood Institute (grant T32HL160522).

## Disclosures Statement

J.B. Strom reports research grants from the National Heart, Lung, and Blood Institute (1R01HL169517, 1R01HL173998), Anumana, Philips Healthcare, EVERSANA Lifesciences, and Bracco Diagnostics; consulting for Bracco Diagnostics, Edwards Lifesciences, Philips Healthcare, General Electric Healthcare, and EVERSANA, and is a member of the scientific advisory boards for Ultromics, HeartSciences, Bristol Myers Squibb, Alnyam, and EchoIQ. The other author had no conflicts to declare.
